# Gut microbiome diversity detected by high-coverage 16S and shotgun sequencing of paired stool and colon sample

**DOI:** 10.1038/s41597-020-0427-5

**Published:** 2020-03-16

**Authors:** Joan Mas-Lloret, Mireia Obón-Santacana, Gemma Ibáñez-Sanz, Elisabet Guinó, Miguel L. Pato, Francisco Rodriguez-Moranta, Alfredo Mata, Ana García-Rodríguez, Victor Moreno, Ville Nikolai Pimenoff

**Affiliations:** 1 Oncology Data Analytics Program, Catalan Institute of Oncology (ICO), Barcelona, Spain; 2 Colorectal Cancer Group, ONCOBELL Program, Bellvitge Institute of Biomedical Research (IDIBELL), Barcelona, Spain; 3 Consortium for Biomedical Research in Epidemiology and Public Health (CIBERESP), Barcelona, Spain; 4 Gastroenterology Department, Bellvitge University Hospital-IDIBELL, Hospitalet de Llobregat, Barcelona, Spain; 5 Cancer Epigenetics and Biology Program (PEBC), Bellvitge Biomedical Biomedical Research Institute (IDIBELL), Barcelona, Catalonia Spain; 6 Digestive System Service, Moisés Broggi Hospital, Sant Joan Despí, Spain; 7 Endoscopy Unit, Digestive System Service, Viladecans Hospital-IDIBELL, Viladecans, Spain; 8 Department of Clinical Sciences, Faculty of Medicine, University of Barcelona, Barcelona, Spain; 9 National Cancer Center Finland (FICAN-MID) and Karolinska Institute, Stockholm, Sweden

**Keywords:** Diagnostic markers, Microbiome, Classification and taxonomy, DNA sequencing

## Abstract

The gut microbiome has a fundamental role in human health and disease. However, studying the complex structure and function of the gut microbiome using next generation sequencing is challenging and prone to reproducibility problems. Here, we obtained cross-sectional colon biopsies and faecal samples from nine participants in our COLSCREEN study and sequenced them in high coverage using Illumina pair-end shotgun (for faecal samples) and IonTorrent 16S (for paired feces and colon biopsies) technologies. The metagenomes consisted of between 47 and 92 million reads per sample and the targeted sequencing covered more than 300 k reads per sample across seven hypervariable regions of the 16S gene. Our data is freely available and coupled with code for the presented metagenomic analysis using up-to-date bioinformatics algorithms. These results will add up to the informed insights into designing comprehensive microbiome analysis and also provide data for further testing for unambiguous gut microbiome analysis.

## Abstract (summary)

**Table:** 

Measurement(s)	genome •rRNA_16S
Technology Type(s)	DNA sequencing
Factor Type(s)	sex •age •Smoking •Weight •Height •Diet •Medication
Sample Characteristic - Organism	Homo sapiens
Sample Characteristic - Environment	feces •colon

Machine-accessible metadata file describing the reported data: 10.6084/m9.figshare.11902236

## Background & Summary

The gut microbiome is highly dynamic and variable between individuals, and is continuously influenced by factors such as individual’s diet and lifestyle^1,2^, as well as host genetics^3^. Next generation sequencing (NGS) has greatly enhanced our understanding of the human microbiome, as these techniques allow researchers to investigate variation in diversity and abundance of bacteria in a culture-independent manner. Recent developments in bioinformatics have permitted the identification of thousands of novel bacterial and archaeal species and strains identified in human and non-human environments through metagenome assembly^4–6^. For colorectal cancer (CRC), recent large-scale studies have revealed specific faecal microbial signatures associated with malignant gut transformations, although the causal role of gut bacterial ecosystem in CRC development is still unclear^7,8^.

The 16S small subunit ribosomal gene is highly conserved between bacteria and archaea, and thus has been extensively used as a marker gene to estimate microbial phylogenies^9^. The 16S rRNA gene contains nine hypervariable regions (V1-V9) with bacterial species-specific variations that are flanked by conserved regions. Hence, the amplification of 16S rRNA hypervariable regions can be used to detect microbial communities in a sample typically down to the genus level^10^, and species-level assignments are also possible if full-length 16S sequences are retrieved^11^.

However, conserved regions are not entirely identical across groups of bacteria and archaea, which can have an effect on the PCR amplification step. Notably, among the conserved regions of the 16S gene, central regions are more conserved, suggesting that they are less susceptible to producing bias in PCR amplification^12^. Furthermore, an *in silico* study has shown that the V4-V6 regions perform better at reproducing the full taxonomic distribution of the 16S gene^13^. In another study, a constructed mock sample was sequenced by IonTorrent technology, demonstrating that the V4 region (followed by V2 and V6-V7) was the most consistent for estimating the full bacterial taxonomic distribution of the sample^14^. In addition, other methodological factors such as the actual primer sequence, sequencing technology and the number of PCR cycles used may impact on microbiome detection when using 16S sequencing. However, the relative ratios in taxonomic abundance have been shown to be consistent regardless of the experimental strategy used^15^.

Beyond 16S sequencing, shotgun metagenomics allows not only taxonomic profiling at species level^16,17^, but may also enable strain-level detection of particular species^18^, as well as functional characterization and *de novo* assembly of metagenomes^19^. Moreover, a plethora of new computational methods and query databases are currently available for comprehensive shotgun metagenomics analysis^20^. However, shotgun metagenomics is more expensive than 16S sequencing and may not be feasible when the amount of host DNA in a sample is high^21^. Nevertheless, provided sufficient sequencing coverage, taxonomic profiling of shotgun metagenomes is rather robust and mostly depends on the input DNA quality and bioinformatics analysis tools^22^. Taken together, 16S and shotgun microbiome profiles from the same samples are not entirely the same, but rather represent the relative microbiome composition captured by each methodological approach^23–26^. In agreement, comparative studies have already revealed that faecal, rectal swab and colon biopsy samples collected from the same individuals usually produce differential microbiome structures although consistent relative taxon ratios and particular core profiles are also detected^27^.

In this study, we characterized the gut microbiome signature of nine participants with paired feacal and colon tissue samples. Our data shows a high concordance between different sequencing methods and classification algorithms for the full microbiome on both sample types. However, clear deviations depending on the sample, method, genomic target and depth of sequencing data were also observed, which warrant consideration when conducting large-scale microbiome studies.

Accompanying this dataset, we also provide the full source code for the bioinformatics analysis, available and thoroughly documented on a GitLab repository. We expect that this annotated, high-quality gut microbiome dataset will provide useful insights for designing comprehensive microbiome analyses in the future, as well as be of use for researchers wishing to test their analysis bioinformatics pipelines.

## Methods

### Subjects and sampling

The COLSCREEN study is a cross-sectional study that was designed to recruit participants from the Colorectal Cancer Screening Program conducted by the Catalan Institute of Oncology. This program invites men and women aged 50–69 to perform a biennial faecal immunochemical test (FIT, OC-Sensor, Eiken Chemical Co., Japan). Patients with a positive test result (≥20 g Hb/g faeces) are referred for colonoscopy examination. A detailed description of the screening program is provided elsewhere^28,29^. Exclusion criteria are as follows: gastrointestinal symptoms; family history of hereditary or familial colorectal cancer (2 first-degree relatives with CRC or 1 in whom the disease was diagnosed before the age of 60 years); personal history of CRC, adenomas or inflammatory bowel disease; colonoscopy in the previous five years or a FIT within the last two years; terminal disease; and severe disabling conditions.

Participants provided written informed consent and underwent a colonoscopy. A week prior to colonoscopy preparation, participants were asked to provide a faecal sample and store it at home at − 20 °C. The day of the colonoscopy, participants delivered the faecal sample. Participants also delivered a self-administered risk-factor questionnaire where they had to report antibiotics, probiotics and anti-inflammatory drugs intake in the previous months (Table 1). Patients reporting any antibiotics or probiotics intake one month prior to sampling were not included in this study.

**Table 1:** Clinical descriptives. Colorectal cancer risk-factor information. Former smoker indicates non-smoker for the last 12 months prior sampling. User consumed non-steroidal anti-inflammatory drugs (NSAIDs) in the 12 months prior sampling.

Sample ID	Sex	Age	Weight (kg)	Height (cm)	Smoking	Red meat (g/day)	Processed meat (g/day)	Vegetables (g/day)	Alcohol (g/day)	NSAIDS use	Family history CRC
AE1235	M	62	64	164	Current	NA	NA	NA	NA	No	No
AE1236	F	67	62	148	Never	19.1	3.7	280.4	0	Yes	No
AE1237	F	63	63	155	Former	NA	NA	NA	NA	Yes	No
AE1238	M	61	73	172	Current	5.8	14.7	264.3	720.1	Yes	Yes
AE1239	F	54	69	166	Current	8.6	8.5	182.5	196.7	Yes	No
AE1240	M	63	83	168	Never	49	0.8	197.9	142.7	No	No
AE1241	F	67	74	160	Never	19.9	6.6	109.7	265	No	No
AE1242	F	67	65	152	Never	NA	NA	NA	NA	No	No
AE1243	F	55	85	160	Never	13	0.8	113.3	557.8	No	No

All stool samples were stored in − 80 °C, while colonic mucosa biopsy samples were retrieved during the colonoscopy. Four biopsies of normal tissue of each colon segment (4 of ascending colon, 4 of transverse colon, 4 of descending colon, and 4 of rectum) were obtained. If a tumour or a polyp was biopsied or removed, a biopsy was obtained if the endoscopist considered it possible. Subsequently, biopsy samples were immediately transferred to RNAlater (Qiagen) and stored at − 80 °C. One biopsy of normal tissue from ascending colon was selected from each of nine individuals and used in this study.

Colonic lesions were classified according to “European guidelines for quality assurance in CRC”^30^. For the present study, we selected patients with no lesions in the colonoscopy, patients with intermediate-risk lesions (3–4 tubular adenomas measuring <10 mm with low-grade dysplasia or as ≥1 adenoma measuring 10–19 mm) and with high-risk lesions (≥5 adenomas or ≥1 adenoma measuring ≥20 mm). We analysed 18 biological samples (9 faecal samples and 9 colon tissue samples) from 9 participants: n = 3 negative colonoscopy, n = 3 high-risk lesions, n = 3 intermediate-lesions) (Table 2). Our CRC screening programme follows the Public Health laws and the Organic Law on Data Protection. All procedures performed in the study involving data from human participants were in accordance with the ethical standards of the institutional research committee, and with the 1964 Helsinki Declaration and its later amendments or comparable ethical standards. The protocol of the study was approved by the Bellvitge University Hospital Ethics Committee, registry number PR084/16.

**Table 2:** Clinical characteristics of the samples and DNA yields. HRA = high-risk adenoma; IRA = intermediate-risk adenoma; neg = healthy colon.

Sample	Sex	Age	FIT result	Condition	DNA (stool, *μ*g)	DNA (tissue, *μ*g)
AE1235	Male	62	−	HRA	4.3	9.1
AE1236	Female	67	−	neg	3.0	15.2
AE1237	Female	63	+	HRA	4.2	31.6
AE1238	Male	61	−	IRA	9.8	15.4
AE1239	Female	54	+	neg	5.2	11.4
AE1240	Male	63	−	neg	3.5	9.3
AE1241	Female	68	+	IRA	5.4	6.5
AE1242	Female	67	+	IRA	6.5	13.6
AE1243	Female	55	+	HRA	2.4	17.1

### DNA extraction and sequencing

Total faecal DNA was extracted using the NucleoSpin Soil kit (Macherey-Nagel, Duren, Germany) with a protocol involving a repeated bead beating step in the sample lysis for complete bacterial DNA extraction. Total DNA from the snap-frozen gut epithelial biopsy samples was extracted using an in-house developed proteinase K (final concentration 0.1 *μ*g/*μ*L) extraction protocol with a repeated bead beating step in the sample lysis. All extracted DNA samples were quantified using Qubit dsDNA kit (Thermo Fisher Scientific, Massachusetts, USA) and Nanodrop (Thermo Fisher Scientific, Massachusetts, USA) for sufficient quantity and quality of input DNA for shotgun and 16S sequencing. DNA yields from the extraction protocols are shown in Table 2.

Metagenomics sequencing libraries were prepared with at least 2 *μ*g of total DNA using the Nextera XT DNA sample Prep Kit (Illumina, San Diego, USA) with an equimolar pool of libraries achieved independently based on Agilent High Sensitivity DNA chip (Agilent Technologies, CA, USA) results combined with SybrGreen quantification (Thermo Fisher Scientific, Massachusetts, USA). The indexed libraries were sequenced in one lane of a HiSeq 4000 run in 2 × 150 bp paired-end reads, producing a minimum of 50 million reads/sample at high quality scores. In total 92.15% of the base calls of the whole sequencing run had a quality score Q30 or higher (i.e. an error rate of 1 in 1000).

Targeted 16S sequencing libraries were prepared using Ion 16S Metagenomics Kit (Life Technologies, Carlsbad, USA) in combination with Ion Plus Fragment Library kit (Life Technologies, Carlsbad, USA) and loaded on a 530 chip and sequenced using the Ion Torrent S5 system (Life Technologies, Carlsbad, USA). The protocol was designed for microbiome analysis using Ion torrent 510/520/530 Kit-chef template preparation system (Life Technologies, Carlsbad, USA) and included two primer sets that selectively amplified seven hypervariable regions (V2, V3, V4, V6, V7, V8, V9) of the 16S gene. At least 10 ng of total DNA was used for 16S library preparation and re-amplified using Ion Plus Fragment Library kit for reaching the minimum template concentration. Equimolar pool of libraries were estimated using Agilent High Sensitivity DNA chip (Agilent Technologies, CA, USA). Library preparation and 16S sequencing was performed with the technological infrastructure of the Centre for Omic Sciences (COS).

### Bioinformatics analysis

Bioinformatics analysis was performed by running in-house pipelines. Shotgun reads were first introduced into a pipeline including removal of human reads and quality control of samples. High quality reads resulting from this pipeline were further analysed under three different approaches: taxonomic classification, functional classification and *de novo* assembly. Additionally, we subsampled high quality shotgun reads to analyse the loss of observed alpha diversity when a lower sequencing depth is reached.

Targeted 16S sequencing reads, on the other hand, were first subjected to a pipeline which identifies variable regions and separates them accordingly. Further denoising and classification analyses were performed separately for each 16S variable region as explained in the following sections.

### Removal of human reads

Prior to submission of the raw sequence data to the European Nucleotide Archive (ENA), human reads were removed from the metagenome samples in order to follow legal privacy policies. Raw reads were aligned to the human genome (GRCh38) using Bowtie2 with options –very-sensitive-local and -k 1. A FASTQ file was then generated from reads which did not align (carrying SAM flag 12) using Samtools. These FASTQ files were deposited to the ENA.

### Shotgun reads quality control

Shotgun samples were quality controlled using FASTQC. Accordingly, sequences were deduplicated using clumpify from the BBTools suite, followed by quality trimming (PHRED > 20) on both ends and adapter removal using BBDuk. Read pairs where one read had a length lower than 75 bases were discarded. Results of this quality control pipeline are shown in Table 3.

**Table 3:** Quality control. Numbers indicate the amount of original microbial paired-end reads and the amount of paired-end reads passing quality control, as well as percentages of read pairs excluded due to duplication or quality and adapter trimming.

Sample	Microbial	High quality	Deduplicated (%)	Trimmed (%)
AE1235	27,510,304	19,991,742	7.42	19.91
AE1236	45,050,043	29,097,088	12.47	22.94
AE1237	25,720,634	18,745,351	7.78	19.34
AE1238	34,831,431	25,727,431	7.78	18.36
AE1239	36,353,427	25,946,121	8.15	20.47
AE1240	31,699,249	23,225,137	8.08	18.65
AE1241	34,083,370	24,830,987	8.04	19.11
AE1242	31,592,814	23,239,834	7.77	18.67
AE1243	23,476,326	17,887,436	7.80	16.01

### Shotgun taxonomic and functional profiling

Pre-processed paired-end shotgun sequences were classified using three different classifiers: Kraken2 (a k-mer matching algorithm), MetaPhlan2 (a marker-gene mapping algorithm) and Kaiju (a read mapping algorithm). These three softwares were chosen to cover the three main algorithms used in taxonomic classification^20^.

**Fig. 1:**
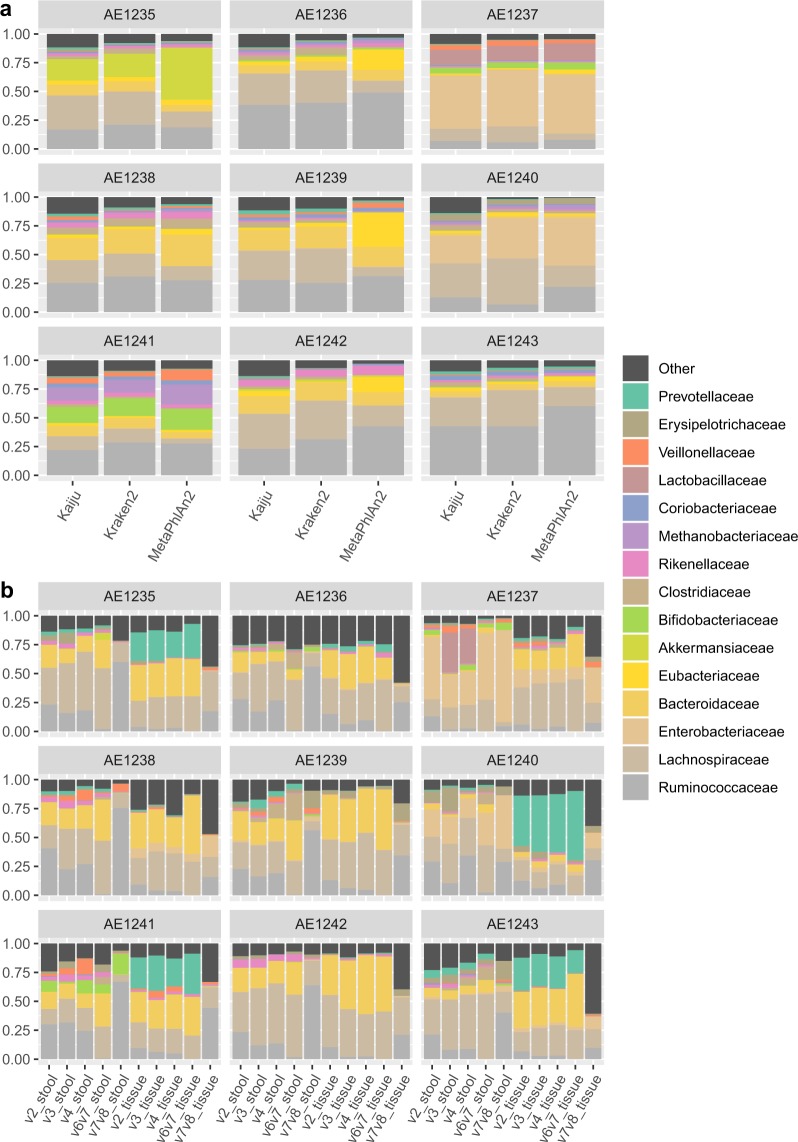
Taxonomic classification of samples at family level. (**a**) Classification of shotgun samples using three different classifiers. (**b**) Classification of 16S sequences, split by region and source material, using DADA2 and IdTaxa.

Kraken2 was run against a reference database containing all RefSeq bacterial and archaeal genomes (built in May 2019) with a 0.1 confidence threshold. Following classification by Kraken, Bracken was used to re-estimate bacterial abundances at taxonomic levels from species to phylum using a read length parameter of 150. MetaPhlAn2 was run using default parameters on the mpa_v20_m200 marker database. Kaiju was run against the Progenomes database (built in February 2019) using default parameters. Corresponding taxonomic profiles at family level are shown in Fig. 1a.

Functional profiling of the concatenated metagenomic paired-end sequences was performed using the HUMAnN2 pipeline with default parameters, obtaining gene family (UniRef90), functional groups (KEGG orthogroups) and metabolic pathway (MetaCyc) profiles. ChocoPhlAn and UniRef90 databases were retrieved in October 2018.

### De novo assembly

High quality metagenomic reads were assembled using metaSPADES with default parameters and binned into putative metagenome assembled genomes (MAGs) using metaBAT. checkM was used to check the quality of MAGs and filter them to comply with strict quality requirements (completeness > 90%, contamination < 5%, number of contigs < 300 %, N50 > 20,000). A total of 112 high quality MAGs were assembled from the nine high-coverage metagenomes and assigned a species-level taxonomy using PhyloPhlAn2. Assembled species shared by at least two of the nine samples are listed in Table 4.

**Table 4:** Metagenome Assembled Genomes (MAGs). Summary of high quality MAGs present in at least two samples (see times observed).

Phylum	Family	Species name	Completeness (%)	Genome size (Mb)	N50 (Kb)	Times observed
Actinobacteria	Coriobacteriaceae	Collinsella aerofaciens	95–100	2.1–2.2	67–72	2
Bacteroidetes	Bacteroidaceae	Bacteroides uniformis	96–97	4.2–4.5	75–117	2
Bacteroidetes	Prevotellaceae	Paraprevotella clara	92–97	3.2–3.4	24–55	2
Bacteroidetes	Rikenellaceae	Alistipes putredinis	92–98	2.0–2.3	61–110	5
Euryarchaeota	Methanobacteriaceae	Methanobrevibacter smithii	95–100	1.6–1.9	76–189	3
Firmicutes	Clostridiaceae	Clostridium sp CAG 127	91–97	2.4–2.6	53–240	3
Firmicutes	Clostridiaceae	Clostridium sp CAG 217	96–97	1.9–2.0	257–320	2
Firmicutes	Clostridiaceae	Clostridium sp L2 50	94–99	2.4–2.6	60–162	2
Firmicutes	Clostridiaceae	Clostridium sp	97–98	2.5–2.7	33–75	3
Firmicutes	Erysipelotrichaceae	Holdemanella SGB6796	94–96	2.1–2.2	25–89	2
Firmicutes	Eubacteriaceae	Eubacterium sp CAG 202	99	2.1–2.3	53–76	2
Firmicutes	Eubacteriaceae	Eubacterium sp CAG 251	99	1.8–1.9	53–143	3
Firmicutes	Lachnospiraceae	Coprococcus eutactus	96	2.6–2.7	22–59	2
Firmicutes	Lachnospiraceae	Dorea longicatena	95–99	2.4–3.2	28–54	2
Firmicutes	Lachnospiraceae	Eubacterium rectale	97–99	2.2–2.8	22–91	5
Firmicutes	Lachnospiraceae	Fusicatenibacter saccharivorans	96–97	2.7–2.9	42–82	3
Firmicutes	Lachnospiraceae	Roseburia sp CAG 45	96–98	2.6–2.7	63–138	3
Firmicutes	Ruminococcaceae	Faecalibacterium prausnitzii	91–99	2.1–2.5	28–123	4
Firmicutes	Ruminococcaceae	Faecalibacterium sp CAG 74	98–99	2.8–3.0	40–133	3
Firmicutes	Ruminococcaceae	Gemmiger formicilis	94–97	2.3–2.7	25–89	2
Firmicutes	Ruminococcaceae	Ruminococcus bromii	98–99	1.9–2.0	28–40	2
Firmicutes	Ruminococcaceae	Ruminococcus sp	91–99	2.3–2.7	24–107	4
Firmicutes	Ruminococcaceae	Ruminococcus torques	92–95	2.2–2.3	24–61	2
Verrucomicrobia	Akkermansiaceae	Akkermansia muciniphila	98	2.8–2.9	105–325	2

### Generation of lower coverage pseudo-samples

Pseudo-samples of lower coverage were generated *in silico* using the reformat tool from the BBTools suite. Five samples were created at 15 M, 10 M, 5 M, 2.5 M, 1 M, 500 K, 100 K and 50 K read pairs coverage.

Pseudo-samples were then classified using Kraken2 and HUMAnN2. From this classification, Shannon index alpha diversity profiles were computed at the species, genus and phylum level, as well as UniRef90, KO and MetaCyc pathways level using the R package vegan.

### Splitting 16S samples by region

As the Ion 16S Metagenomics Kit contains several primers in the PCR mix, the resulting FASTQ files contained sequencing reads belonging to different variable regions. Hence, an in-house Python program was written in order to identify the variable region(s) present in each read. Then, FASTQ files were stratified into new subfiles where all sequences contained belonged to the same region.

First, we positioned the 16S conserved regions^12^ in the *E. coli* str. K-12 substr. MG1655 16S reference gene (SILVA v.132 Nr99 identifier U00096.4035531.4037072) as well as the corresponding variable region positions^10^. Regions 5 and 7 were truncated to match the reference *E. coli* sequence. Each sequencing read was then assigned into its corresponding variable region by mapping.

Analysis of the regions covered in our samples revealed a prevalence of V3, followed by V4, V2, V6-V7 and V7-V8 (Table 5). For each sample, each set of sequences from the same variable region(s) was subsequently extracted from the original FASTQ files with an in-house Python script (code available).

**Table 5:** Targeted 16S data. Percentage of 16S reads covering each region in the corresponding sample.

		Total	V2	V3	V4	V6-V7	V7-V8	Other
Faeces	AE1235	739819	3.2	40.2	14.3	21.6	18.8	1.9
	AE1236	450511	2.9	43.6	15.0	20.6	16.0	2.0
	AE1237	767495	4.1	36.0	14.4	17.6	24.8	3.2
	AE1238	740788	3.6	38.5	14.5	20.6	21.0	1.8
	AE1239	997171	5.9	36.1	14.2	24.2	17.6	2.0
	AE1240	458735	2.4	39.0	13.5	17.3	24.8	2.9
	AE1241	590541	3.5	40.0	14.0	19.6	21.0	1.9
	AE1242	467170	3.4	37.8	14.7	19.7	22.6	1.9
	AE1243	386045	3.3	41.0	14.6	21.0	18.1	2.0
Tissue	AE1235	321453	4.3	61.1	14.2	15.1	4.5	0.9
	AE1236	621908	8.3	46.8	16.7	18.7	8.7	0.8
	AE1237	726770	8.2	43.8	17.5	18.4	11.0	1.1
	AE1238	735109	7.4	42.3	18.7	17.8	11.5	2.3
	AE1239	577808	6.8	49.1	16.5	20.7	6.2	0.8
	AE1240	601785	9.5	42.3	19.1	21.4	6.6	1.0
	AE1241	649667	7.9	45.7	17.3	24.9	3.4	0.8
	AE1242	589330	5.4	50.4	16.6	23.2	3.6	0.9
	AE1243	447223	7.0	48.0	19.4	16.7	8.1	0.8

### 16S denoising and taxonomic binning

16S sequences were denoised following the standard DADA2 pipeline with adaptations to fit our single-end read data. For this analysis, reads spanning different regions, obtained in the previous step, were introduced into the pipeline as different input files. Taxonomic classification of the high-quality sequences was performed using IdTaxa included in the DECIPHER package. A summary of quality estimates of the DADA2 pipeline is shown in Table 6. Taxonomic assignment at family level by region and source material is shown in Fig. 1b.

**Table 6:** DADA2 results. Total amount of reads entering the pipeline and passing all the quality controls are indicated, as well as percentages of reads filtered in each step.

Source	Sample ID	Region	Input	Output	Filtered (%)	Denoised (%)	Chimeras (%)
Stool	AE1235	v2	23675	18409	16.27	2.99	2.98
	AE1235	v3	297069	204763	14.80	0.26	16.01
	AE1235	v4	105530	72361	26.79	1.17	3.47
	AE1235	v6v7	160139	118416	14.27	1.74	10.04
	AE1235	v7v8	139431	102517	23.41	1.19	1.87
	AE1236	v2	13177	10091	20.25	3.00	0.17
	AE1236	v3	196436	148363	12.94	0.30	11.22
	AE1236	v4	67353	46528	28.87	1.27	0.78
	AE1236	v6v7	92647	71073	13.38	1.78	8.13
	AE1236	v7v8	72100	55878	18.57	1.26	2.66
	AE1237	v2	31697	22779	21.13	2.02	4.98
	AE1237	v3	276040	201847	14.04	0.34	12.50
	AE1237	v4	110375	82233	19.16	0.98	5.36
	AE1237	v6v7	135004	91005	16.34	1.28	14.98
	AE1237	v7v8	190178	126317	18.27	0.72	14.59
	AE1238	v2	26631	21196	14.94	3.29	2.18
	AE1238	v3	285027	206419	12.46	0.37	14.74
	AE1238	v4	107172	80701	19.20	1.72	3.77
	AE1238	v6v7	152748	111924	11.94	2.03	12.76
	AE1238	v7v8	155514	111841	18.88	1.02	8.19
	AE1239	v2	58730	46507	14.39	1.74	4.68
	AE1239	v3	359574	251532	15.33	0.24	14.48
	AE1239	v4	141973	103323	21.22	1.19	4.82
	AE1239	v6v7	241379	173393	11.71	1.53	14.93
	AE1239	v7v8	175774	130720	18.40	1.03	6.20
	AE1240	v2	11200	8381	16.34	4.73	4.10
	AE1240	v3	179016	123229	16.20	0.47	14.50
	AE1240	v4	62106	47971	18.49	1.67	2.60
	AE1240	v6v7	79313	50315	17.02	3.24	16.30
	AE1240	v7v8	113851	83697	18.19	1.64	6.65
	AE1241	v2	20533	15287	18.88	3.23	3.43
	AE1241	v3	236319	164152	15.45	0.40	14.68
	AE1241	v4	82470	62916	20.12	1.63	1.96
	AE1241	v6v7	115842	83998	13.58	2.75	11.16
	AE1241	v7v8	124095	89112	19.74	1.26	7.19
	AE1242	v2	16093	12590	16.98	3.80	0.98
	AE1242	v3	176603	116141	17.49	0.39	16.36
	AE1242	v4	68441	51756	19.43	1.91	3.03
	AE1242	v6v7	91881	67003	16.06	2.16	8.86
	AE1242	v7v8	105442	81780	15.77	1.39	5.28
	AE1243	v2	12651	9882	16.73	3.60	1.56
	AE1243	v3	158164	112772	13.44	0.37	14.89
	AE1243	v4	56432	40641	24.63	1.38	1.97
	AE1243	v6v7	81212	57972	13.32	2.92	12.38
	AE1243	v7v8	69949	52240	19.07	2.26	3.99
Tissue	AE1235	v2	13680	10741	18.41	1.69	1.39
	AE1235	v3	196304	144394	11.75	0.23	14.46
	AE1235	v4	45755	35944	20.18	0.42	0.84
	AE1235	v6v7	48383	39295	15.96	0.67	2.16
	AE1235	v7v8	14445	11208	21.16	0.97	0.28
	AE1236	v2	51480	42622	15.80	0.50	0.91
	AE1236	v3	291280	226960	11.57	0.16	10.35
	AE1236	v4	103690	79166	22.58	0.21	0.86
	AE1236	v6v7	116437	101656	11.56	0.19	0.94
	AE1236	v7v8	53800	40664	20.83	0.57	3.01
	AE1237	v2	59739	48980	14.92	0.61	2.47
Tissue	AE1237	v3	318023	228121	12.38	0.16	15.73
	AE1237	v4	126872	94309	24.77	0.14	0.76
	AE1237	v6v7	133901	111136	13.67	0.33	3.00
	AE1237	v7v8	79930	58141	23.29	0.52	3.46
	AE1238	v2	54373	43554	16.29	0.82	2.79
	AE1238	v3	311029	227554	13.57	0.24	13.03
	AE1238	v4	137377	106679	20.87	0.32	1.16
	AE1238	v6v7	130753	112947	11.57	0.26	1.79
	AE1238	v7v8	84391	62281	23.08	0.60	2.52
	AE1239	v2	39380	32759	14.47	0.86	1.49
	AE1239	v3	283485	206573	11.36	0.16	15.61
	AE1239	v4	95146	74237	20.74	0.24	1.00
	AE1239	v6v7	119410	102233	11.41	0.35	2.63
	AE1239	v7v8	35846	27409	19.80	1.07	2.66
	AE1240	v2	57468	45978	16.02	0.77	3.20
	AE1240	v3	254594	182648	13.86	0.23	14.17
	AE1240	v4	115056	89991	20.65	0.13	1.01
	AE1240	v6v7	129027	106387	15.19	0.33	2.03
	AE1240	v7v8	39782	30472	20.14	0.62	2.63
	AE1241	v2	51322	42185	16.15	0.85	0.80
	AE1241	v3	297068	231915	12.17	0.10	9.66
	AE1241	v4	112313	85034	22.84	0.29	1.16
	AE1241	v6v7	161575	140379	12.25	0.20	0.67
	AE1241	v7v8	22036	16680	20.72	1.37	2.22
	AE1242	v2	31761	26112	16.67	1.04	0.07
	AE1242	v3	297138	233551	12.07	0.12	9.21
	AE1242	v4	97818	76855	20.07	0.17	1.18
	AE1242	v6v7	136577	116654	12.59	0.26	1.74
	AE1242	v7v8	21025	16087	21.35	0.86	1.28
	AE1243	v2	31236	25427	16.92	1.12	0.56
	AE1243	v3	214598	161786	12.69	0.26	11.66
	AE1243	v4	86913	69844	18.09	0.45	1.10
	AE1243	v6v7	74483	65530	10.91	0.53	0.58
	AE1243	v7v8	36358	28409	18.68	1.01	2.18

### Statistical analysis

For the statistical analysis of the bacterial abundance data, we used compositional data analysis methods^31^.

Count matrices of the classified taxa were subjected to central log ratio (CLR) transformation after removing low-abundance features and including a pseudo-count. Here, we used the codaSeq.filter, cmultRepl and codaSeq.clr functions from the CodaSeq and zCompositions packages. Principal components analysis (PCA) biplots were generated from the central log ratios using the prcomp function in R.

## Data Records

The raw sequence data generated in this work were deposited into the European Nucleotide Archive (ENA). Faecal metagenomic sequences are available under accession PRJEB33098^32^. Faecal 16S sequences are available under accession PRJEB33416^33^ and tissue 16S sequences are available under accession PRJEB33417^34^. Human sequences were removed from whole shotgun samples as previously described prior to the ENA submission.

## Technical Validation

Prior to analysis, shotgun sequencing reads were subject to quality and adapter trimming as previously described. Moreover, reads were deduplicated to avoid compositional biases caused by PCR duplicates. Quality control and denoising of 16S reads was performed within the DADA2 denoising pipeline and not as an independent data processing step.

In order to validate the 16S variable region assignment, we selected reads that were assigned to a species by the assignSpecies function in DADA2, which searches for unambiguous full-sequence matches in the SILVA database. These pre-processed 16S reads were aligned to a full length 16S gene from those species in the SILVA database (version 132, gene codes shown in Table 7). The reads mapped consistently in regions within the 16S gene in agreement with the variable region assigned by our pipeline. That is, each read was assigned between the start and end loci reported in Table 7, and corresponding to the estimated 16S variable region for the particular microbe species genomes. These results suggest that our read level 16S region assignment was largely correct.

**Table 7:** 16S alignment validation. Region(s) covered by 16S reads with exact matches to the SILVA database. The first column represents the region(s) called by our pipeline, while the third and fourth show the exact matching positions in the SILVA database. This shows consistency between the variable region called by our pipeline and the expected position it occupies along the 16S gene. SILVA IDs: *B. fragilis*: FQ312004.3243020.3244552; *B. vulgatus*: CP000139.2183533.2185042; *F. nucleatum*: AE009951.530422.531923; *R. gnavus*: AZJF01000012.178214.179732.

Region	Species	Start	End
v2	*F. nucleatum*	134	389
v2	*R. gnavus*	108	362
v2	*B. vulgatus*	110	364
v2	*B. fragilis*	108	361
v3	*B. vulgatus*	330	540
v3	*B. fragilis*	327	537
v4	*F. nucleatum*	531	818
v4	*R. gnavus*	500	788
v4	*B. vulgatus*	522	810
v6v7	*F. nucleatum*	944	1207
v6v7	*R. gnavus*	917	1177
v6v7	*B. vulgatus*	936	1194
v6v7	*B. fragilis*	933	1193

**Table 8:** Bioinformatic tools. Software versions and related resources.

Software	Use	Version	
Bowtie2	Human reads mapping	2.3.4	^36^
Samtools	Extraction of non-human reads	1.8	^37^
FASTQC	Reads quality assessment	0.11.7	^38^
Clumpify	Removal of duplicate reads	38.26	^39^
BBDuk	Quality and adapter trimming	38.26	^39^
Kraken	Taxonomic classification of shotgun reads	2.0.8-beta	^40^
Bracken	Re-estimation of taxonomic profiles	2.2	^41^
MetaPhlAn2	Taxonomic classification of shotgun reads	2.7.8	^42^
Kaiju	Taxonomic classification of shotgun reads	1.6.3	^43^
HUMAnN2	Functional profiling of shotgun reads	0.11.1	^44^
metaSPADES	Metagenomic assembly	3.13.1	^45^
metaBAT	Binning of scaffolds	2.12.1	^46^
checkM	Bins quality assessment	1.0.12	^47^
PhyloPhlAn2	Taxonomic classification of bins	0.35	^48^
Reformat	Generation of lower coverage samples	38.26	^39^
DADA2 (R)	Denoising of 16S reads	1.10.1	^49^
IdTaxa (R)	Taxonomic classification of 16S sequences	2.10.1	^50^
vegan (R)	Computation of alpha diversity	2.5.3	^51^
zCompositions (R)	Compositional data analysis	0.99.3	^52^
CoDaSeq (R)	Compositional data analysis (https://github.com/ggloor/CoDaSeq)	1.2.0	

**Fig. 2:**
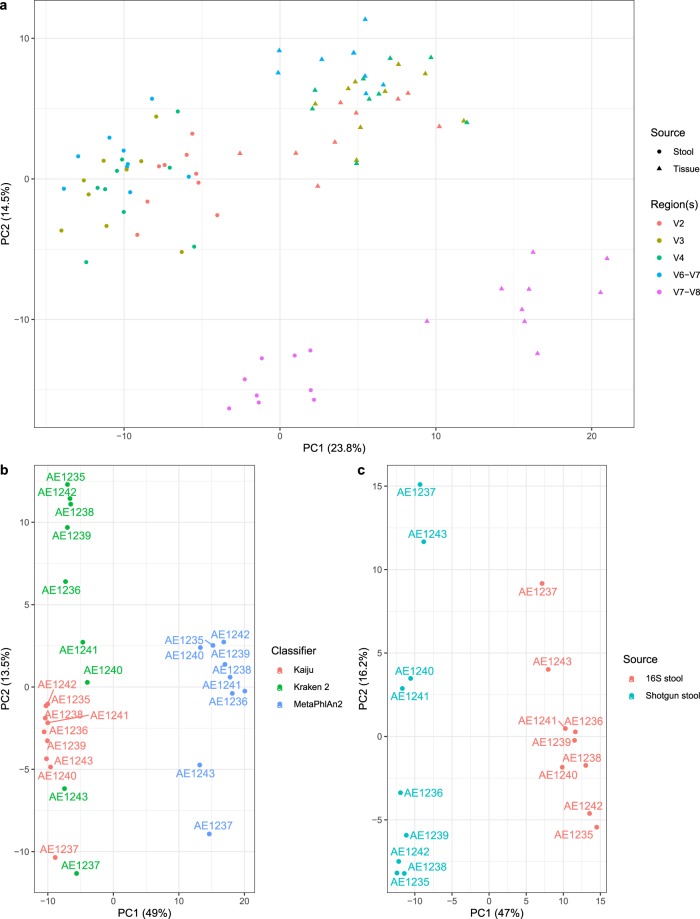
Ordination. Principal components analysis of the datasets after central log ratio transformations of the family-level classifications. (**a**) 16S data, where each sample data was stratified by region and source material. (**b**) Shotgun data, classified using Kraken2, Kaiju and MetaPhlAn2. (**c**) 16S data from faeces (only V4 region) and shotgun data (classified using Kraken2).

To define the taxonomic structure of the microbiome, we compared three different classifier algorithms which are based on full genome k-mer matching (Kraken2), protein-level read alignment (Kaiju) or gene specific markers (MetaPhlAn2) (Fig. 1a). A common core microbiome structure was observed regardless of the taxonomic classifier method. However, particular deviations in relative abundance were observed between these methods. To estimate the microbiome community structure differences, we performed a PCA of CLR-transformed data, which revealed a clear clustering by the taxonomic classification method (Fig. 2b). Importantly, however, Kraken2 and Kaiju family-level classifications clustered samples in the same order along the second component, which likely reflects consistency in classification despite of the method used.

Both variable regions analysed and the source material (faeces or tissue) revealed differential distributions of the bacterial taxa (Fig. 1b). Indeed, when analysing CLR-transformed taxonomic profiles, samples clustered mostly by source material (Fig. 2a). Notably, the V7-V8 data showed the largest deviation in principal components from all other variable regions (Fig. 2a).

Altogether, a clear difference in community structure was observed between 16S and shotgun sequences from the same faecal sample (Fig. 2c). Regardless, samples were displayed in the same order on the second component, which indicated consistency of the detected microbial signature.

**Fig. 3:**
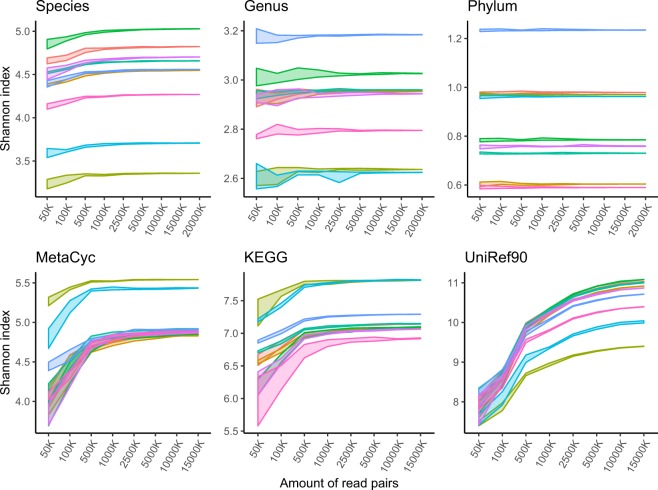
Alpha diversity. Shannon index was calculated at different taxonomic levels (species, genus, phylum, top row) as classified by Kraken2 and functional (gene families: UniRef90, functional groups: KEGG orthogroups and metabolic pathways: MetaCyc, bottom row) levels as classified by HUMAnN2 by number of read pairs. Five random samples were created at each level.

Finally, we subsampled original high quality reads for lower coverage and computed alpha diversity at different taxonomic and functional levels in order to estimate the sequencing depth necessary to capture the observed microbial diversity in a given sample (Fig. 3).

These alpha diversity profiles demonstrated a gradual drop in diversity as sequencing coverage decreased. This drop in coverage was more noticeable in features with higher diversity, particularly at species level or when using gene families (UniRef90). Altogether, in the case of species, sequencing coverages as low as 1 million read pairs appeared to capture the taxonomic diversity present in a sample, in line with previous findings^35^. In this study, we demonstrate that our high-coverage dataset from nine participants sustained sufficient sequencing depth to capture the majority of the known bacterial taxa and functional groups present in the samples.

## Usage Notes

For reproducibility purposes, sequencing data was deposited as raw reads. However, human sequencing reads were removed from the dataset prior to uploading in order to prevent participants’ identification. Thus, reads need to be trimmed and, if necessary, deduplicated, before being reutilized.

For 16S data, reads have been uploaded without any manipulation. Hence, reads from different variable regions are present in the same FASTQ file. We suggest researchers to run the reads classification scripts in order to choose variable regions for the analysis. Following that, reads will still need to be quality controlled, either directly or by denoising algorithms such as DADA2.

## Data Availability

Software versions used are listed in Table 8. Code for sequence quality control and trimming, shotgun and 16S metagenomics profiling and generation of figures in this paper is freely available and thoroughly documented at https://gitlab.com/JoanML/colonbiome-pilot. This repository includes instructions for the analysis and reproduction of the figures on this paper from the publicly available samples, as well as pipelines used for the analysis. This repository is arranged in folders, each containing a README: • qc: Scripts for quality control and preprocessing of samples • analysis_shotgun: Scripts to run softwares for metagenomics analysis • regions_16s: In-house scripts for splitting IonTorrent reads into new FASTQ files • analysis_16s: DADA2 pipeline adapted to this dataset • assembly: Scripts to run the assembly, binning and quality control software • figures: Scripts used to generate the figures in this manuscript • shannon_index_subsamples: Scripts used to compute alpha diversity in subsampled FASTQs
